# Uniformity under *in vitro* conditions: Changes in the phenotype of cancer cell lines derived from different medulloblastoma subgroups

**DOI:** 10.1371/journal.pone.0172552

**Published:** 2017-02-23

**Authors:** Petr Chlapek, Karel Zitterbart, Leos Kren, Lenka Filipova, Jaroslav Sterba, Renata Veselska

**Affiliations:** 1 Laboratory of Tumor Biology, Department of Experimental Biology, Faculty of Science, Masaryk University, Brno, Czech Republic; 2 International Clinical Research Center, St. Anne’s University Hospital and Faculty of Medicine, Masaryk University, Brno, Czech Republic; 3 Department of Pediatric Oncology, University Hospital Brno and Faculty of Medicine, Masaryk University, Brno, Czech Republic; 4 Department of Pathology, University Hospital Brno and Faculty of Medicine, Masaryk University, Brno, Czech Republic; University of Navarra, SPAIN

## Abstract

Medulloblastoma comprises four main subgroups (WNT, SHH, Group 3 and Group 4) originally defined by transcriptional profiling. In primary medulloblastoma tissues, these groups are thought to be distinguishable using the immunohistochemical detection of β-catenin, filamin A, GAB1 and YAP1 protein markers. To investigate the utility of these markers for *in vitro* studies using medulloblastoma cell lines, immunoblotting and indirect immunofluorescence were employed for the detection of β-catenin, filamin A, GAB1 and YAP1 in both DAOY and D283 Med reference cell lines and the panel of six medulloblastoma cell lines derived in our laboratory from the primary tumor tissues of known molecular subgroups. Immunohistochemical detection of these markers was performed on formalin-fixed paraffin-embedded tissue of the matching primary tumors. The results revealed substantial divergences between the primary tumor tissues and matching cell lines in the immunoreactivity pattern of medulloblastoma-subgroup-specific protein markers. Regardless of the molecular subgroup of the primary tumor, all six patient-derived medulloblastoma cell lines exhibited a uniform phenotype: immunofluorescence showed the nuclear localization of YAP1, accompanied by strong cytoplasmic positivity for β-catenin and filamin A, as well as weak positivity for GAB1. The same immunoreactivity pattern was also found in both DAOY and D283 Med reference medulloblastoma cell lines. Therefore, we can conclude that various medulloblastoma cell lines tend to exhibit the same characteristics of protein marker expression under standard *in vitro* conditions. Such a finding emphasizes the importance of the analyses of primary tumors in clinically oriented medulloblastoma research and the urgent need to develop *in vitro* models of improved clinical relevance, such as 3D cultures and organotypic slice cultures.

## Introduction

Approximately 10% of all pediatric cancer-related deaths are caused by medulloblastoma, an embryonal neuroectodermal tumor of the cerebellum [1]. Current mechanisms for clinical stratification in medulloblastoma include the criteria of age, metastatic disease and extent of surgical resection. Prognostication using histopathological subgrouping is based on the recognition of highly aggressive large cell/anaplastic variants or favorable desmoplastic/extensive nodular variants in infants [2,3]. Comprehensive studies of the medulloblastoma genome, epigenome and transcriptome have led to the current concept of four molecular subgroups: WNT Group, SHH Group, and two non-WNT/non-SHH groups: Group 3 and Group 4 [4–10].

From a clinical point of view, the amount of genomic and molecular data gained over the last few years encourages optimism that improved risk stratification based on biological prognostic markers and new molecular targets will improve the outcomes in medulloblastoma. However, the transition from bench to bedside and from knowledge to its applications is hampered by the lack of robust and reliable tests that could be easily used in routine practice.

The highly complex technologies that generated the progress of "medulloblastomics" are not widely used for diagnostic purposes at this moment. Diagnostic tests for routine subgrouping will be probably based on methylation profiling employing formalin-fixed tissues [11,12]. For immunohistochemistry (IHC), attempts have been made to discriminate among the WNT, SHH, and Group 3/Group 4 subgroups using reactivity for β-catenin, filamin A, GAB1 and YAP1 [13]. An overview of this classification is given in Table 1. However, this panel of antibodies has not yet gained routine diagnostic status because of the lack of strong reproducibility in independent laboratories and patients cohorts, mainly due to the technical aspects of immunohistochemistry. Thus, immunohistochemistry should be combined with molecular genetic methods for precise diagnosis [14,15].

**Table 1:** Subgrouping of medulloblastomas based on the evaluation of IHC immunoreactivity for selected protein markers [13].

Molecular group	Immunoreactivity			
	β-catenin	Filamin A	GAB1	YAP1
SHH	Cytoplasmic	Cytoplasmic	Cytoplasmic	Nuclear + Cytoplasmic
WNT	Nuclear + Cytoplasmic	Cytoplasmic	Negative	Nuclear + Cytoplasmic
Non-SHH/WNT	Cytoplasmic	Negative	Negative	Negative

The basic biological models for medulloblastoma translational research are genetically engineered mouse models and cell culture [16]. However, there is a general lack of validated cell lines according to subgroup; a few of the most common cell lines are regarded as group specific, based mostly on the detection of driver gene mutations or amplifications [16–20].

Here, we employed a unique panel of six medulloblastoma cell lines derived in our laboratory from the primary tumor tissues of known molecular subgroups to determine whether the immunodetection pattern of markers suggested for diagnostic IHC methods is also suitable for group assignment in cell lines. The expression of β-catenin, filamin A, GAB1 and YAP1 was evaluated in these cell lines and in formalin-fixed paraffin-embedded (FFPE) tissue samples of the corresponding tumors from which these cell lines were derived. We hypothesized that the relevant phenotypes of medulloblastoma cells under *in vitro* conditions might differ.

## Materials and methods

### Tumor samples

Six tumor samples were included in this study. These samples were taken from the patients (5 males, 1 female; age range: 1–22 years) surgically treated for medulloblastoma. The tumor tissue was subdivided into several parts. Fresh tumor samples were processed for primary cultures as described below, and FFPE samples were used for immunohistochemical (IHC) analyses. A description of the cohort of patients included in this study is provided in Table 2. The molecular subgroups of the primary tumor tissues were determined as part of a previous global cohort medulloblastoma study [7].

**Table 2:** Description of the patient cohort and patient-derived cell lines.

Tumor sample	Gender	Age	Time of biopsy	Histopathological subgroup	Primary cell line
1	M	1	DG	Extensive nodularity	MBL-12
2	M	15 (22*)	REC	Large cell MBL	MED01
3	M	5	DG	Classic	MBL-06
4	F	11	DG	Desmoplastic	MBL-02
5	M	2	DG	Classic	MBL-13
6	M	7	DG	Classic	MBL-03

Gender: M, male; F, female. Age at the time of diagnosis (years); asterisk indicates age at the time of recurrence of the disease. Time of biopsy: DG, diagnostic; REC, recurrence of the disease.

### Cell lines

Cell lines were derived from the respective tumor tissues according to a previously described protocol [21]. All of these cell lines were derived with the written informed consent obtained for our previous research project (IGA MZCR NR/9125-4), and they can be used for other research purposes if they are handled in the laboratory in an anonymous or coded manner. The previous research project was approved by the Research Ethics Committee of the School of Medicine, Masaryk University, Brno, Czech Republic (Approval No. 23/2005). According to Czech legal and ethical regulations governing the use of human biological material for research purposes, a new ethical assessment of this research study is not necessary. Assignment of these cell lines to the respective tumor samples is given in Table 2. In addition to these patient-derived cell lines, two other MBL cell lines–D283 Med (ATCC HTB-185™) and DAOY (ATCC HTB-186™)–were used as reference cell lines in this study.

### Cell culture

The DAOY cell line was maintained in Dulbecco's modified Eagle's medium (DMEM) supplemented with 10% fetal calf serum (FCS), 1% non-essential amino acids, 2 mM glutamine, and antibiotics: 100 IU/ml penicillin and 100 μg/ml streptomycin (all purchased from PAA Laboratories, Linz, Austria). All of the other cell lines were maintained in DMEM supplemented with 20% FCS, 2 mM glutamine, and antibiotics as specified above. All of the cell lines were cultivated under standard conditions at 37°C in an atmosphere of 95% air and 5% CO_2_.

### Immunohistochemistry for tumor tissue analysis

Representative sections from archival FFPE tumor samples were analyzed by immunohistochemistry (IHC). Formalin-fixed, paraffin-embedded 4-μm-thick sections from both tissue microarray blocks were cut, deparaffinized with pure xylene for 3× 5 min, washed in 96% alcohol for 3× 5 min and finally rinsed with distilled water. Endogenous peroxidase was inactivated by 3% H_2_O_2_ in methanol for 10 min, the samples were then rinsed with distilled water, and antigen retrieval was performed (incubation in citrate buffer of pH 6.0 at 98°C for 20 min and then cooling for 20 min and washing in PBS for 3× 5 min) followed by incubation with primary antibodies. The incubations were performed in a wet chamber for 1 h at room temperature followed by rinsing with PBS for 3× 5 min. The EnVision^+^System streptavidin-biotin peroxidase detection system (Dako, Glostrup, Denmark) was used in accordance with the manufacturer's instructions in the wet chamber at room temperature for 45 min, followed by rinsing with PBS and then visualization using 3,3'diaminobenzidine as a substrate (Sigma-Aldrich, St. Louis, MO, USA). Nuclei were counterstained using Gill's hematoxylin for 1 min followed by bluing in water for 2–3 min for optimal results. Following dehydration in a series of up-concentrated ethanol baths and clearing in xylene, the preparations were mounted onto Entelan™ (Entelan Microscopy, Karlsruhe, Germany). All of the antibodies used in this protocol are described in Table 3. Positive and negative controls were evaluated in each IHC run. A section of liver tissue retrieved from the files of the Department of Pathology served as a positive control for β-catenin (S1 Fig), YAP1 and filamin A positivity (nuclear and cytoplasmic, respectively) was observed in the endothelial cells within tumor samples which served as the positive internal controls (S1 Fig). Breast carcinoma tissue, also retrieved from the files of Department of Pathology served as a positive control for GAB1 (S1 Fig). Negative controls consisted of slides run without the primary antibodies. An Olympus BX45 microscope (Olympus Optical, Tokyo, Japan) equipped with an Olympus DP50 digital camera was used for the evaluation of IHC staining and to capture the micrographs. Olympus Viewfinder Lite™ software was used to process the images. The evaluation was performed separately by two qualified histopathologists. In the rare event of discrepancy, the consensus was reached by subsequent simultaneous evaluation and discussion at the multi-headed microscope.

**Table 3:** Antibodies used in this study.

**Primary antibodies**						
**Antigen**	**Type / Host**	**Clone**	**Manufacturer**	**Dilution**		
				**IHC**	**IF**	**WB**
β-catenin	Monoclonal / Rb	Clone 14	BD Biosciences	1: 100	1: 100	1: 1000
Filamin A	Monoclonal / Mo	10R-F113A	Fitzgerald Industries	1: 50	1: 100	1: 1000
GAB1	Monoclonal / Mo	1A7	Abcam	1: 50	1: 100	1: 500
YAP1	Monoclonal / Mo	sc-101199	Santa Cruz Biotechnology	1: 50	1: 100	1: 1000
β-actin	Polyclonal / Rb	AC-15	Sigma-Aldrich	–	–	1: 10 000
α-tubulin	Monoclonal / Mo	TU-01	Exbio	–	1: 100	–
**Secondary antibodies **						
**Host**	**Specificity**	**Conjugate**	**Manufacturer**	**Dilution**		
				**IHC**	**IF**	**WB**
Goat	anti-RbIgG	Alexa Fluor 488	Life Technologies	–	1: 200	–
Goat	anti-MoIgG	Alexa Fluor 488	Life Technologies	–	1: 200	–
Horse	anti-RbIgG	HRP	Cell Signaling	–	–	1: 5000
Horse	anti-MoIgG	HRP	Cell Signaling	–	–	1: 5000

Rb, rabbit; Mo, mouse; HRP, horseradish peroxidase

### Immunofluorescence for cell line analysis

The cell suspensions were seeded onto glass coverslips and were grown under standard conditions for 24 h. The cells were then washed in phosphate-buffered saline (PBS), fixed with 3% para-formaldehyde (Sigma) in PBS at room temperature for 20 min, and permeabilized with 0.2% Triton X-100 (MP Biomedicals, Santa Ana, CA, USA) in PBS at room temperature for 1 min. The cells were subsequently washed in PBS and incubated with 2% bovine serum albumin (PAA) for 10 min to block nonspecific binding of the secondary antibodies. In the next step, the cells were treated with primary antibodies at 37°C for 1 h and then were washed three times in PBS. The corresponding secondary antibody conjugated with Alexa Fluor 488 was applied under the same conditions. All of the antibodies used in this protocol are described in Table 3; a mouse monoclonal anti-α-tubulin served as the positive control (S2 Fig). Finally, the cells were counterstained by Hoechst 33342 (Sigma) for 10 min and were mounted onto glass slides in Vectashield mounting medium (Vector Laboratories, Burlingame, CA, USA). The specimens were observed using an Olympus BX-51 fluorescence microscope. The intensity of the immunostaining (immunoreactivity) of detected proteins was evaluated in 200 cells at randomly selected discrete areas of each specimen. Micrographs were captured with a CCD camera Olympus DP72 and were processed using software Cell^P 3.4 (Olympus).

### Immunoblotting for cell line analysis

Whole-cell extracts were loaded onto polyacrylamide (Sigma) gels– 6% for the detection of Filamin A and 8% for others–electrophoresed, and blotted onto polyvinylidene difluoride membranes (Bio-Rad Laboratories, Hercules, California). The membranes were blocked with 5% nonfat milk in PBS with 0.1% Tween 20 (Sigma) and then were incubated primary in blocking solution at 4°C overnight. After rinsing with PBS-T, the membranes were incubated with the corresponding secondary antibody at room temperature for 60 min. All of the antibodies used in this protocol are also described in Table 3. Each step was followed by at least three 10 min washes in PBS-T. ECL-Plus detection was performed according to the manufacturer’s instructions (Amersham, GE Healthcare, UK).

## Results

### Subgrouping using the diagnostic IHC method in FFPE tissues confirmed the results from expression profiling or revealed an inconclusive pattern of immunoreactivity

**Fig 1:**
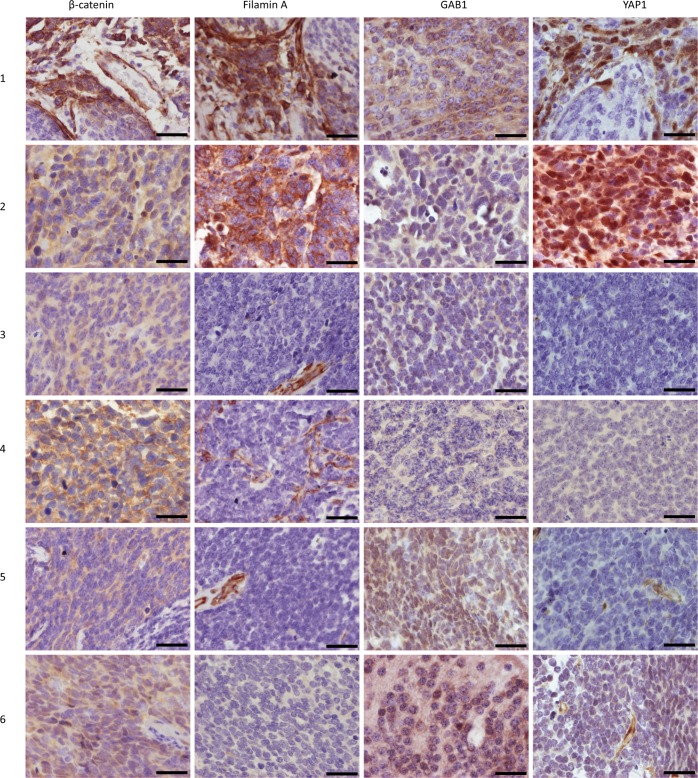
Immunohistochemical detection of selected protein markers in tumor samples. Expression of β-catenin, filamin A, GAB1 and YAP1 in medulloblastoma tissue samples (1–6) as detected by immunohistochemistry. Bars, 100 μm.

IHC detection of markers using anti-β-catenin, anti-filamin A, anti-GAB1 and anti-YAP1 antibodies was employed in FFPE tissue samples (Fig 1, Table 4). The immunoreactivity was consistent with an SHH pattern (filamin A-positive, GAB-positive and YAP-positive) in two samples (No. 1 and 2) and with a non-SHH/WNT pattern (filamin A-, GAB- and YAP-negative) in the other two samples (No. 3 and 4). Nevertheless, the immunoreactivity of the two remaining medulloblastomas (No. 5 and 6) did not correspond to any of the categories as described within the classification system by Ellison et al. [13]: positive immunolabeling for GAB and/or YAP was found, but labeling for filamin produced negative results.

**Table 4:** Analysis of the expression and intracellular localization of selected protein markers in tumor samples and corresponding cell lines.

Tumor sample	Cell line	Expression profiling Tumor	IHC Tumor	Expression in tumor cells							
				(Intracellular localization: nuclear |cytoplasmic)							
				β-catenin		Filamin A		GAB1		YAP1	
				IHC	IF	IHC	IF	IHC	IF	IHC	IF
				Tumor	Cell line	Tumor	Cell line	Tumor	Cell line	Tumor	Cell line
1	MBL-12	SHH	SHH	- | +++	- |+++	-|+++	- |+++	++|++	- | +	++|++	+++|++
2	MED01	SHH	SHH	- | +++	- |+++	-|+++	- |++	++|+	- | -	+++|++	+++|++
3	MBL-06	Group 3	Group 3/4	- | +++	- |+++	- | -	- |+++	++|++	- | ++	- | -	+++|++
4	MBL-02	Group 4	Group 3/4	- | +++	- |+++	- | -	- |++	- | -	- | +	- | -	+++|++
5	MBL-13	Group 3	UD	- | +++	- |+++	- | -	- |+++	+++|++	- | +	- | -	+++|++
6	MBL-03	Group 4	UD	- | +++	- |+++	- | -	- |++	+++|+++	- | -	++ |-	+++|++
DAOY reference cell line				NA	- |++	NA	- |++	NA	+ | ++	NA	++|+
D283 Med reference cell line				NA	- |++	NA	- |++	NA	+ | +	NA	++|+

In the tumor samples analyzed by IHC, the percentage of positive tumor cells was categorized into four levels as follows: − (0%), + (1–10%), ++ (11–50%), and +++ (51–100%). The intracellular localization (nuclear or cytoplasmic) of the respective marker is indicated. In cell lines analyzed by IF, the pattern of staining was homogeneous and the intensity of immunostaining (immunoreactivity) was categorized into three levels: +, weak; ++, medium; +++, strong. The medulloblastoma groups revealed by expression profiling [7] and by immunodetection according to previously published criteria [12] are also indicated. UD, undefinable; NA, not available.

After unblinding the molecular subgroup [7], assignment concordance was revealed for both SHH samples. The medulloblastomas with an ambiguous IHC pattern were genetically group 3 (sample No. 5) and group 4 (sample No. 6), respectively.

### MBL cell lines showed a uniform phenotype regardless of the tumor classification

Two methods–immunofluorescence and immunoblotting–were used for the detection of four molecular markers (β-catenin, filamin A, GAB1 and YAP1) in patient-derived cell lines to compare the results with those obtained from the corresponding tumor samples on the protein level. Furthermore, two reference medulloblastoma cell lines, DAOY and D283 Med, were included in these analyses.

**Fig 2:**
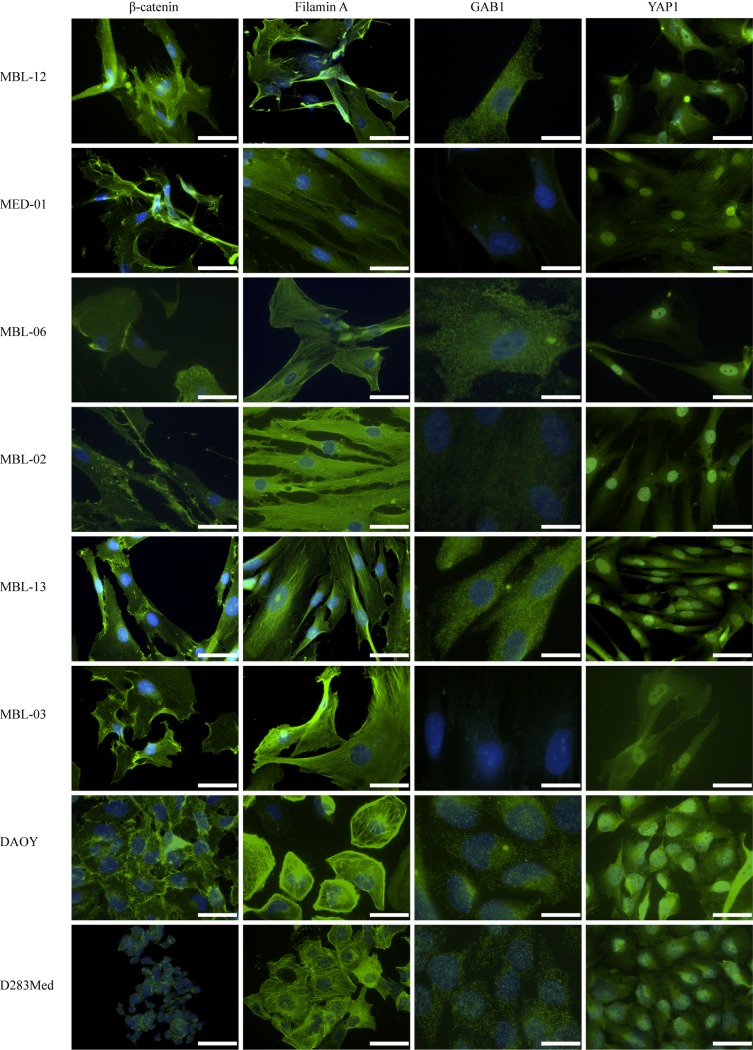
Immunofluorescence detection of selected protein markers in corresponding cell lines. Expression of β-catenin, filamin A, GAB1 and YAP1 in medulloblastoma cell lines as detected by IF. Each marker was visualized by indirect immunofluorescence using Alexa 488-conjugated secondary antibody (green); nuclei were counterstained with DAPI (blue). Bars, 50 μm (β-catenin, filamin A, YAP1) or 20 μm (GAB1).

The results from immunofluorescence detection are summarized in Table 4, and representative micrographs of the labeling patterns are also given (Fig 2). The percentage of positive cells was nearly 100% in all cases; therefore, we evaluated only the immunoreactivity–i.e., the intensity of immunostaining. In all of the examined cell lines, we found nuclear localization of YAP1 as well as cytoplasmic localization of β-catenin and filamin A. For all of these three markers, the immunoreactivity varied from weak to strong. GAB1 protein was found in the cytoplasm of four patient-derived cell lines only with weak immunoreactivity; MED01 and MBL-03 were detected as GAB1-negative. Both reference cell lines showed a uniform labeling pattern, which was in accordance with that of the patient-derived cell lines.

**Fig 3:**
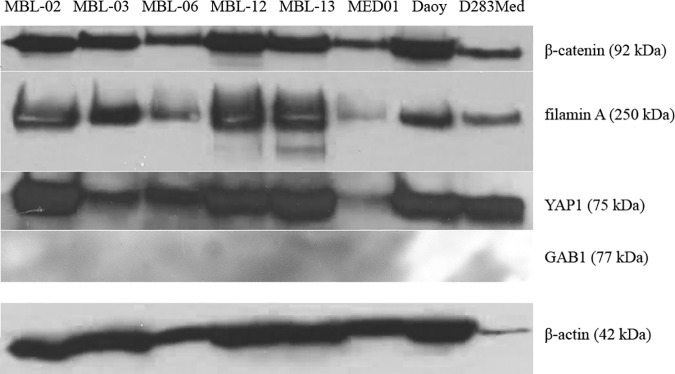
Immunoblot analysis of selected protein markers in medulloblastoma cell lines. Expression of β-catenin, filamin A, GAB1 and YAP1 in patient-derived and reference medulloblastoma cell lines as detected by immunoblotting. β-actin served as a loading control.

In all of the examined cell lines, the almost uniform expression of β-catenin, filamin A and YAP1 was verified by immunoblotting as summarized in Fig 3. The GAB1 protein was undetectable by this method, similar to the results obtained by immunofluorescence.

Both immunofluorescence and immunoblotting clearly showed that all six patient-derived cell lines acquired the uniform phenotype under *in vitro* conditions regardless of the original medulloblastoma subgroup classification of the respective tumor tissue. Furthermore, these results fully corresponded to the observed phenotypes of both reference medulloblastoma cell lines, DAOY and D283 Med.

## Discussion

Although cell lines have been successfully used in cancer research for decades and the limitations of such biological models are widely recognized and accepted [22], many *in vitro* studies have presumed–despite *in vitro* selection and heterogeneity–that a cancer cell line preserves the important biological features of cancer cells in the original tumor from which the cell line was derived. This issue was recently discussed for various human solid tumors, including ovarian carcinoma [23,24], breast carcinoma [25], lung carcinoma [26] or thyroid carcinoma [27].

In this study, we investigated the possibilities of using the intracellular protein markers GAB1, β-catenin, filamin A, and YAP1 for the determination of defined molecular subgroups of medulloblastoma under *in vitro* conditions. To compare the phenotype of medulloblastoma cells both *in situ* and after transfer into cell culture, six samples of medulloblastoma tissues together with six corresponding cell lines derived in our laboratory from these tumors were used for this study. DAOY and D283 Med established medulloblastoma cell lines served as reference cell lines for these experiments. To determine the medulloblastoma subgroups in tumor tissue samples, the IHC detection of the four protein markers mentioned above was performed and the achieved results were compared to those obtained by gene expression profiling on the same tumor samples [7]. In cell lines, immunofluorescence and immunoblotting were employed for the detection of the protein markers in question.

Our results revealed substantial divergence in the immunoreactivity pattern of the four medulloblastoma-subgroup-specific protein markers between the primary tumor tissue and matching cell line. Regardless of the molecular subgroup of the primary tumor, all six patient-derived medulloblastoma cell lines exhibited a uniform phenotype: immunofluorescence showed the nuclear localization of YAP1 accompanied by strong cytoplasmic positivity for β-catenin and filamin A, as well as weak positivity for GAB1 (Table 4, Fig 2). The same immunoreactivity pattern was also found in both DAOY and D283 Med reference medulloblastoma cell lines.

These discrepancies in the expression pattern of protein markers between the primary tumor tissues and derived cell lines can be explained from several viewpoints. One of them is the lower sensitivity of the IHC method used for the detection of these markers in tumor tissue than that with immunofluorescence and immunoblotting used in the analysis of the cell lines. The other explanation is based on the specific functions of the marker proteins within the cell and on the influence of the cell culture conditions on these functions.

The cultivation of cells in a monolayer has an apparently strong impact on the cell morphology, organization of the cytoskeleton and on the proteins participating in the formation of cell-cell and cell-substrate adhesions [28]. Such changes can provide an explanation especially of the marked increase in filamin A expression under *in vitro* conditions. Filamins are large actin-binding proteins that stabilize three-dimensional actin network and link the actin filaments to cellular membranes [29], and filamin A is known as a protein involved in the regulation of cell adhesion via interactions with cytoskeletal components and plasma membrane proteins [30]. Because cancer cells in cell culture usually have a rearranged actin cytoskeleton due to the changed cell shape and higher motility under *in vitro* conditions, an increase in filamin A expression is not surprising in such a situation and fully corresponds to the previously published studies on the role of filamin A in cultured cells [31–33]. Very recently, the protumorigenic and antitumorigenic role of filamin A is also discussed regarding its intracellular localization: high levels of filamin A in the cytoplasm are associated with tumor-promoting cell behavior [34].

Similarly, members of the Gab family interact with specific membrane lipids and with signaling proteins encompassing the SH2 and SH3 domains. They typically act as downstream effectors of receptor tyrosine kinase-triggered signal transduction that controls many processes, including the regulation of cell polarity [35], and a decrease in their expression under *in vitro* conditions may be associated with changed cell morphology and polarity. Nevertheless, the increased levels of Gab1 protein were associated with decreased oncogenic potential and with phenotypic changes accompanying monocytic differentiation of murine myeloid cells: increased attachment to the substrate and filopodia formation [36].

Another mechanism is probably responsible for the apparent increase in the expression of YAP1 protein: a higher concentration of oxygen in cell culture than in the tumor bulk *in vivo* may induce the overexpression of proteins involved in the response to oxidative stress. Although YAP1 is known primarily as the oncogene involved in the Hippo tumor suppressor pathway, the induction of YAP1 expression caused by an increase in the intracellular ROS concentration was described in glioblastoma cells [37].

In all of the analyzed cell lines, we found no discrepancy in the expression or localization of β-catenin. β-catenin is known as one of the proteins participating in adherens junctions, and it also plays a key role in the WNT signaling pathway. Thus, the nuclear accumulation of β-catenin is routinely used as a biomarker of the WNT pathway and is typical for the WNT medulloblastoma subgroup [6]. This consistent pattern of β-catenin cytoplasmic localization in all of the cell lines analyzed in our study finally confirms the uniform SHH-group phenotype of these cell lines under *in vitro* conditions, as defined by the described protein markers [13].

A uniform pattern of protein marker expression was observed in all patient-derived cell lines included in this study as well as in two reference cell lines, although the respective primary tumors showed diverse expression patterns belonging to different medulloblastoma subgroups. This phenotype uniformity of our patient-derived cell lines with the DAOY and D283 Med reference cell lines is another very interesting aspect of our results. Because both of these cell lines were previously analyzed using expression profiling, their transcriptomic molecular classification is available but is partly controversial: the DAOY cell line was described consistently as belonging to the SHH group, and the results for the D283 Med cell line were contradictory and varied between groups 3 and 4 [20,38,39]. Nevertheless, the achieved results on the analysis of the Ellison's protein markers showed very similar patterns also in these two reference cell lines corresponding to the SHH-group of medulloblastomas. Therefore, it is obvious that, regardless of the molecular profiles determined by the transcriptomic methods, various medulloblastoma cell lines tend to exhibit the same phenotype under standard *in vitro* conditions.

This finding is in accordance with other recently published studies: substantial discrepancies between primary tumors and related cancer cell lines have already been described using genomic [40,41], transcriptomic [16,42] or proteomic [43] approaches. Furthermore, very similar patterns of expressed multidrug-resistance genes were found within a large group of human cancer cell lines, whereas the differences between the cell line and respective primary tumor were substantial [42]. Finally, a poor overlap and correlation between medulloblastoma primary tumors and related cell lines was also described at the transcriptional level [16].

## Conclusions

Achieved results clearly showed that various medulloblastoma cell lines tend to exhibit the same characteristics of protein marker expression under standard *in vitro* conditions. Because clinically relevant medulloblastoma subgroups are defined exactly based on transcriptional profiles, such a finding emphasizes the importance of analyzing primary tumors rather than cell line model systems in clinically oriented medulloblastoma research. Moreover, the urgent need for the development of *in vitro* models of improved clinical relevance, such as 3D cultures and organotypic slice cultures, is also evident.

## Supporting information

S1 Fig Positive controls for immunohistochemical detection of selected protein markers. Expression of β-catenin in liver tissue, expression of filamin A and YAP1 in endothelial cells within the medulloblastoma tissue, and expression of GAB1 in breast carcinoma tissue as detected by immunohistochemistry. Bars, 100 μm.
(TIF)

S2 Fig Detection of α-tubulin as positive control (green) for the immunofluorescence labeling protocol in medulloblastoma cell lines. Bars, 50 μm.
(TIF)

S3 Fig Complete images of Western blots with molecular weight ladders. (TIF)
