# Supplementary figures and images for: Uniformity under in vitro conditions: Changes in the phenotype of cancer cell lines derived from different medulloblastoma subgroups
Source: PLoS One. 2017 Feb 23;12(2):e0172552. doi: 10.1371/journal.pone.0172552 (PMC5322931; doi:10.1371/journal.pone.0172552)

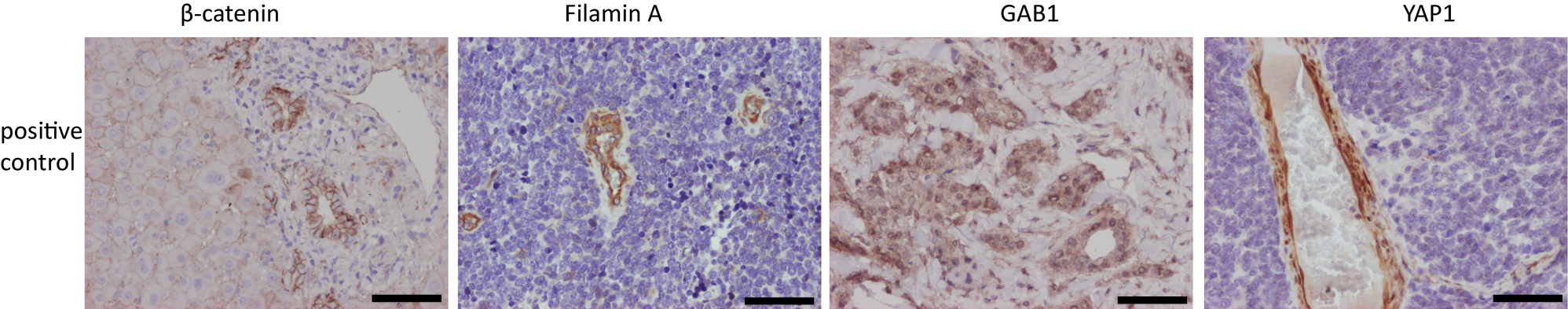

Supplement: S1 Fig — Expression of β-catenin in liver tissue, expression of filamin A and YAP1 in endothelial cells within the medulloblastoma tissue, and expression of GAB1 in breast carcinoma tissue as detected by immunohistochemistry. Bars, 100 μm. (TIF) [file pone.0172552.s001.tif]

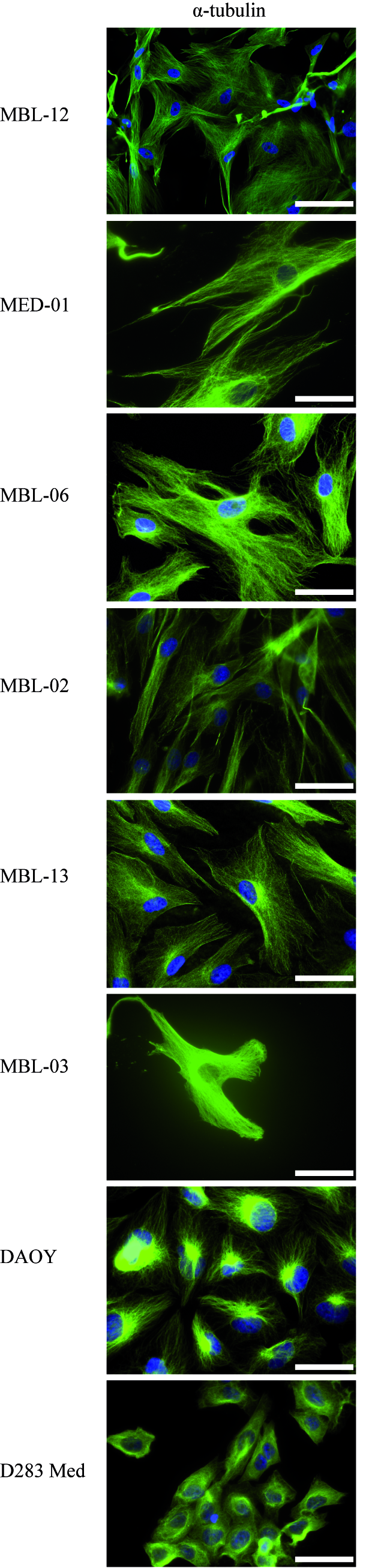

Supplement: S2 Fig — Bars, 50 μm. (TIF) [file pone.0172552.s002.tif]

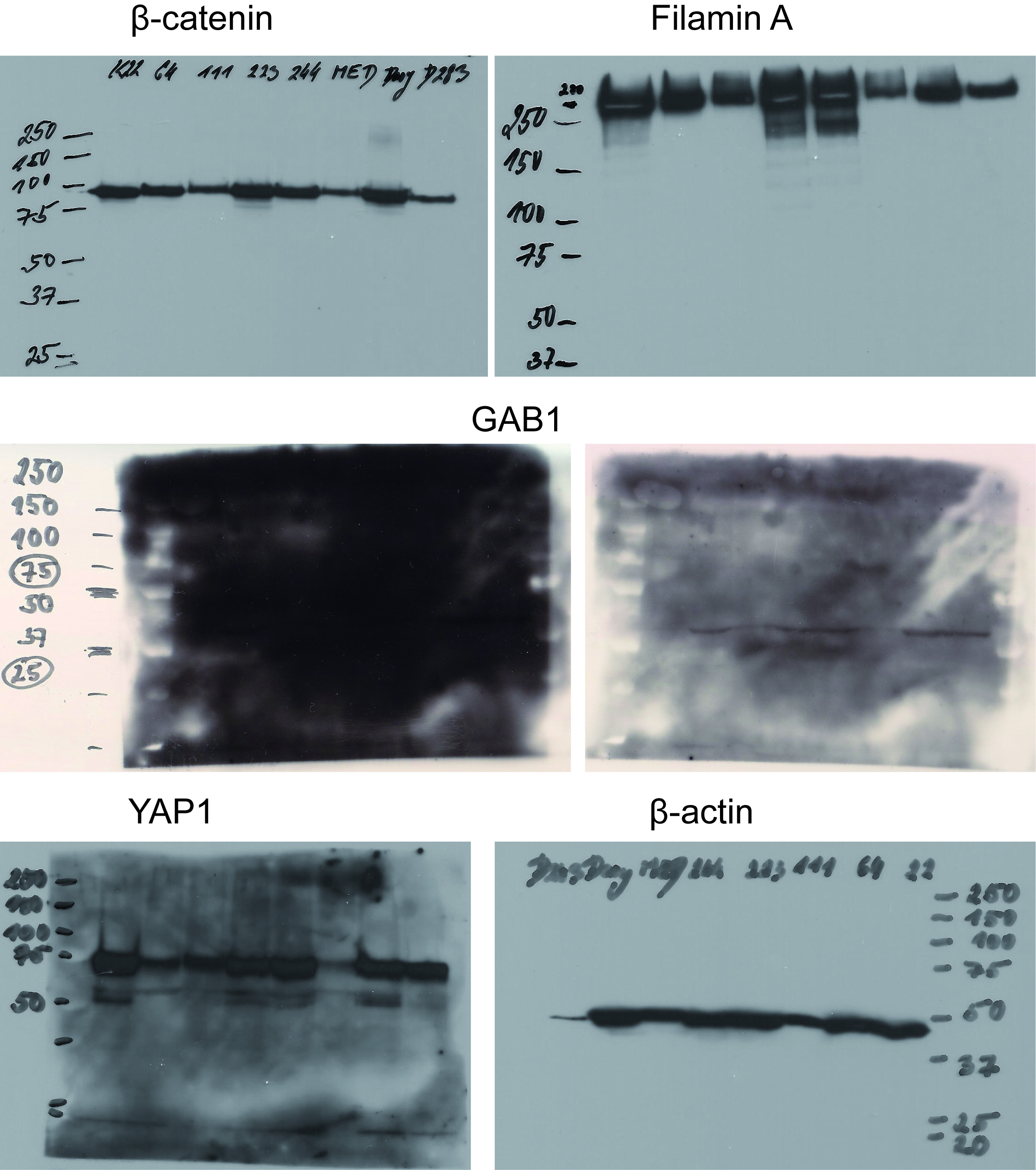

Supplement: S3 Fig — (TIF) [file pone.0172552.s003.tif]
